# Dataset on microplastic concentrations, characteristics, and chemical composition in the marine surface waters of Latvia – the Eastern Gotland basin and the Gulf of Riga

**DOI:** 10.1016/j.dib.2023.108992

**Published:** 2023-02-17

**Authors:** Marta Barone, Natalija Suhareva, Juris Aigars, Ieva Putna-Nimane, Inta Dimante-Deimantovica

**Affiliations:** a Latvian Institute of Aquatic Ecology, 4 Voleru Str., Riga LV-1007, Latvia; b Daugavpils University, The Faculty of Natural Sciences and Mathematics, 1 Parades str., LV-5401, Daugavpils, Latvia

**Keywords:** Plastic pollution, Litter, Baltic sea, Sea surface, FTIR

## Abstract

The dataset provides information on spectroscopically verified microplastics, both particles and fibers, from 44 marine surface water samples of two Baltic Sea sub-basins – the semi-enclosed Gulf of Riga and the Eastern Gotland Basin. Sampling was performed by using Manta trawl with a mesh size of 300 µm. Thereafter, the organic material was digested with sodium hydroxide, hydrogen peroxide and enzymes. Samples were filtered on glass fiber filters and analyzed visually, registering the shape, size, and color of each item. Where feasible, the polymer type was determined using Attenuated Total Reflection Fourier Transform Infrared (ATR-FTIR) spectroscopy method. The number of plastic particles per m^3^ of filtered water was determined. The data presented in this article may be useful for further research on microplastic pollution, meta-analysis and calculation of microplastic flow. Interpretation and analysis of total acquired data on micro debris and microplastics are reported in the article “Occurrence and spatial distribution of microplastics in the surface waters of the Baltic Sea and the Gulf of Riga”.

**Specifications Table**

**Table:** 

Subject	Environmental Science, Pollution
Specific subject area	Occurrence and characterisation of microplastic contamination in marine surface water
Type of data	Table Figure
How data were acquired	Visual analysis was performed using stereomicroscope Leica DM400 B LED fitted with camera DFC 295 and software Leica Application Suite V4.1. For chemical composition analysis Thermo Fisher Scientific Nicolet iS20 spectrometer and OMNIC software was used.
Data format	Raw
Description of data collection	Collection of 44 samples from May to September, 2018. Recording of start and end coordinates of the transect and meteorological parameters (wind speed and direction, wave height, air temperature). Characterization of microplastic particles by shape, size, colour, and detection of polymer. Quantification per one cubic meter of filtered water. Acquisition of microplastic particle images, spectra and reference spectra identified by ATR-FTIR spectroscopy. Quality assurance for sample loss and contamination prevention. Positive controls for determining recovery rates.
Data source location	Institution: Latvian Institute of Aquatic Ecology City: Riga Country: Latvia Latitude and longitude for collected samples: presented in the Table 1. of this paper
Data accessibility	Repository name: Mendeley Data Data identification number: N/A Direct URL to data: http://doi.org/10.17632/x9ptrn83sz.2
Related research article	J. Aigars, M. Barone, N. Suhareva, I. Putna-Nimane, I. Dimante-Deimantovica. Occurrence and spatial distribution of microplastics in the surface waters of the Baltic Sea and the Gulf of Riga. Marine Pollution Bulletin. 172 (2021), 112860, https://doi.org/10.1016/j.marpolbul.2021.112860[1]

## Value of the Data

• The comprehensive original dataset from Baltic Sea 44 coastal and offshore stations in marine surface waters under the jurisdiction of Latvia, what has not been studied before for microplastic pollution, substantially contributes to already existing knowledge base on composition and spatial distribution of spectroscopically verified microplastics encountered in the marine environment,
• The data would be useful for researchers attempting to model spatial distribution of spectroscopically verified microplastics in marine environment and for calibration of existing models,
• Obtained data can also be used as baseline information for further research to identify the differences of microplastic contamination between regions, sampling sites, as well as a reference value to facilitate establishment of environmental quality standards,
• This data set can serve as a base for further research activities attempting to pinpoint sources and transport pathways of microplastics encountered in marine environment.

## 1 Objective

An extensive study investigating the composition and spatial distribution of micro debris and microplastic pollution in the coastal and open sea surface waters of the Gulf of Riga and the Eastern Gotland Basin, Baltic Sea [1] suggested the necessity of follow up studies by arguing that surface water microplastics debris pollution levels might be highly variable depending on different environmental factors, e.g., meteorological conditions and hydrodynamical characteristics distinctive for certain region. However, the available data on this is relatively scarce. With the data made accessible in this article, we aim to contribute to filling the existing knowledge gap and provide detailed information on spectroscopically verified microplastics characteristics and environmental conditions during sampling events for further studies and research activities. We also encourage to use this data for data-driven decision making and as a baseline for assessment of changes in the level of pollution.

## 2 Data Description

**Fig. 1:**
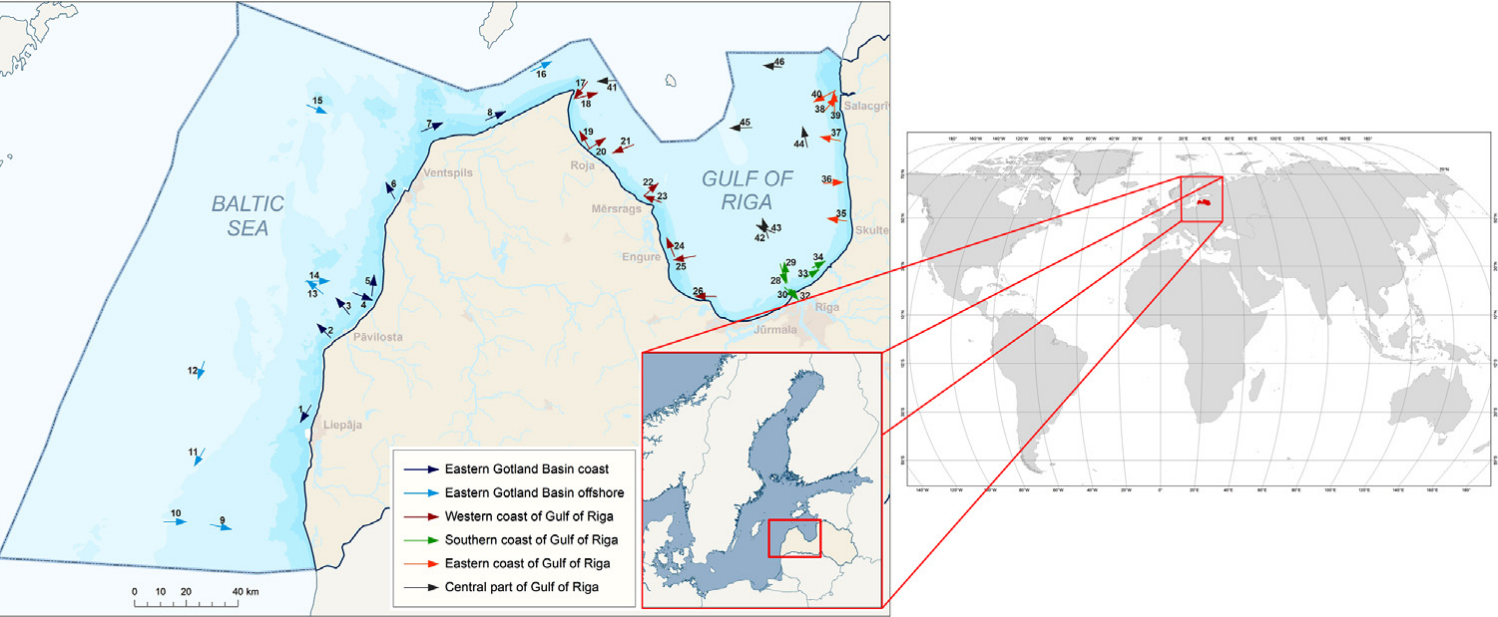
Study area and sampling locations of surface water microplastics in the Eastern Gotland Basin and the Gulf of Riga. Arrows indicate the direction of transect.

The dataset contains information on micro debris and microplastic particle presence and characteristics (shape, size, color and polymer) in 44 samples of marine surface waters under the jurisdiction of Latvia – Eastern Gotland basin and Gulf of Riga. The map showing study area and sampling sites is shown in the Fig. 1. The Table 1 presents the geographical location of each sampling site and related background information, such as transect length, filtered water volume and meteorological parameters.

**Table 1:** Sampling site locations, parameters and meteorological conditions during sampling.

Date	Start coordinates	End coordinates	Transect length, m	Filtered water volume, m^3^	Wind direction	Wind speed, m/s	Wave height, m	Air temperature, C
08.08.2021.	N 56°37.5476 E 20°58.9329	N 56°35.8528 E 20°57.0926	3660	641	SE	5.5	0.2	20.8
28.06.2021.	N 56°52.6576 E 21°05.4406	N 56°54.0992 E 21°02.9134	3700	648	NE	2	0.2	21.2
23.07.2021.	N 56°58.6332 E 21°10.9784	N 56°59.9856 E 21°08.8679	3290	576	W	0.5	0.1	19.3
28.06.2021.	N 57°01.3277 E 21°17.6128	N 57°00.6835 E 21°02.9134	3700	648	SE	4	0.1	-
08.08.2021.	N 57°01.8821 E 21°21.1152	N 57°03.8698 E 21°21.3338	3690	646	SW	5	0.5	25.4
08.08.2021.	N 57°22.2757 E 21°27.9782	N 57°23.4031 E 21°26.7190	2440	427	SW	3.5	0.2	24.6
08.08.2021.	N 57°37.0123 E 21°40.9500	N 57°37.7734 E 21°44.4397	3740	655	SE	4	0.2	24
09.08.2021.	N 57°39.7022 E 22°05.2765	N 57°40.5212 E 22°08.7986	3810	667	SE	9.2	0.3	23.5
22.07.2021.	N 56°12.1866 E 20°26.3145	N 56°11.8383 E 20°29.8987	3760	658	SW	2.5	0.3	24.1
22.07.2021.	N 56°12.5627 E 20°09.1771	N 56°12.6508 E 20°12.8219	3760	658	SW	4	0.3	21.5
21.07.2021.	N 56°27.4020 E 20°19.7521	N 56°25.6067 E 20°18.0872	3740	655	S	3	0.2	21.4
21.07.2021.	N 56°45.3331 E 20°17.8944	N 56°43.3819 E 20°16.7326	3810	667	SW	6	0.5	22.6
23.07.2021.	N 57°02.7716 E 20°59.4386	N 57°04.0060 E 20°56.5163	3730	653	-	0	0.2	20.4
24.07.2021.	N 57°04.0060 E 20°56.5163	N 57°04.0986 E 20°59.6960	3210	562	SW	1.5	0.2	20.4
24.07.2021.	N 57°39.7838 E 20°57.3034	N 57°39.6496 E 20°58.1920	916	160	-	0	0.1	22.5
24.07.2021.	N 57°49.9417 E 22°21.2135	N 57°50.7911 E 22°24.6600	3750	656	N	4	0.3	25.4
09.08.2021.	N 57°45.7493 E 22°39.1997	N 57°44.1022 E 22°37.0255	3740	655	SE	7	0.4	21.6
24.07.2021.	N 57°44.2496 E 22°39.6763	N 57°44.2900 E 22°39.8447	200	35	N	2	0.1	27.1
12.08.2021.	N 57°33.6167 E 22°43.0631	N 57°35.3310 E 22°41.1298	3720	651	SW	13	0.4	16.5
12.08.2021.	N 57°33.4575 E 22°42.7709	N 57°34.5307 E 22°45.8494	3680	644	SW	7.5	0.4	19.1
20.09.2021.	N 57°34.0916 E 22°58.6166	N 57°33.3919 E 22°55.0616	3770	660	S	6.4	0.5	16.6
11.08.2021.	N 57°23.9617 E 23°04.7047	N 57°25.3140 E 23°07.4490	3740	655	SW	6.2	0.5	19.5
10.08.2021.	N 57°23.0785 E 23°08.2310	N 57°23.8612 E 23°04.7272	3790	663	SW	4.3	0.4	20.5
10.08.2021.	N 57°11.4129 E 23°16.3595	N 57°13.3024 E 23°15.0242	3750	656	W	4.3	0.3	23
10.08.2021.	N 57°11.0515 E 23°20.2205	N 57°10.5920 E 23°16.6697	3700	648	S	5.5	0.3	23
10.08.2021.	N 57°03.1111 E 23°28.6882	N 57°03.0935 E 23°24.9762	3750	656	S	7.5	0.3	29.2
18.06.2021.	N 57°07.8000 E 23°58.4000	N 57°06.1000 E 23°59.2000	3260	571	W	6	0.2	-
12.07.2021.	N 57°05.0300 E 23°59.8000	N 57°09.9000 E 23°59.0000	9000	1575	NE	4	0.1	21
07.05.2021.	N 57°04.0100 E 24°00.650	N 57°02.710 E 24°03.550	3800	665	-	-	-	-
12.07.2021.	N 57°03.7000 E 24°01.4000	N 57°02.3000 E 24°04.1000	3770	660	NE	4	0.1	21
18.06.2021.	N 57°07.7000 E 24°08.7000	N 57°08.6000 E 24°11.7000	3480	609	W	6	0.3	-
12.07.2021.	N 57°09.5000 E 24°11.1000	N 57°10.3000 E 24°14.0000	3300	578	NE	4	0.15	21
18.09.2021.	N 57°18.8509 E 24°22.2070	N 57°19.0627 E 24°18.5889	3650	639	S	2.4	0.3	23.1
18.09.2021.	N 57°26.8235 E 24°17.2613	N 57°26.9089 E 24°20.9759	3710	649	SW	6.1	0.1	19.4
18.09.2021.	N 57°35.5892 E 24°19.9819	N 57°36.0878 E 24°16.2021	3870	677	SW	8	0.5	19.1
13.08.2021.	N 57°43.0340 E 24°16.5460	N 57°44.2047 E 24°18.2664	2760	483	NW	5.5	0.3	20.7
19.09.2021.	N 57°43.5423 E 24°18.0398	N 57°45.5420 E 24°18.1732	3680	644	SW	9.6	0.5	19
19.09.2021.	N 57°45.8017 E 24°17.9162	N 57°44.7634 E 24°14.2337	4120	721	SW	8.8	0.8	18.5
20.09.2021.	N 57°47.9191 E 22°53.2276	N 57°47.8322 E 22°49.4371	3750	656	SW	10.1	0.5	17.5
18.06.2021.	N 57°15.2000 E 23°52.6000	N 57°18.0000 E 23°51.0000	5450	954	SW	6	0.15	-
12.07.2021.	N 57°16.5000 E 23°54.1000	N 57°17.2000 E 23°51.0000	3400	595	NE	4	0.15	21
20.09.2021.	N 57°36.1031 E 24°06.7918	N 57°38.0215 E 24°05.9182	3660	641	SW	6.4	0.5	18.8
19.09.2021.	N 57°38.2800 E 23°42.0600	N 57°38.2200 E 23°38.2800	3750	656	SW	9.2	0.7	17.6
20.09.2021.	N 57°51.1474 E 23°54.6829	N 57°51.3146 E 23°50.9321	3720	651	SW	9.2	0.6	17.9

The Table 2 presents total number of micro debris and microplastic particles visually identified in each sample for extended comparability as well as visually identified microplastic particles verified by chemical composition analysis in each sample, and the background airborne contamination registered during samples visual analysis. The concentrations are expressed as the total number of particles and particles per one cubic meter of filtered water.

**Table 2:** Total number and concentration of micro debris (MD) and microplastic (MP) particles and background contamination in samples.

			Number of MD and MP particles				Number of MP particles				
Sub-region	Sample ID	Sample volume m3	Fiber	Non-fiber	Total	MD and MP particle concentrations, items per m^3^	Fiber	Non-fiber	Total	MP particle concentrations, items per m^3^	Background contamination, number of fibers
Eastern Gotland Basin coastal area	**1**	640.5	72	30	102	0.16	4	23	27	0.04	13
	**2**	647.5	58	438	496	0.77	4	403	407	0.63	31
	**3**	575.75	45	56	101	0.18	7	48	55	0.10	34
	**4**	647.5	77	284	361	0.56	1	246	247	0.38	17
	**5**	645.75	101	139	240	0.37	3	113	116	0.18	52
	**6**	427	414	22	436	1.02	4	15	19	0.04	26
	**7**	654.5	124	331	455	0.70	1	303	304	0.46	8
	**8**	666.75	50	8	58	0.09	0	6	6	0.01	9

Eastern Gotland Basin offshore area	**9**	658	128	43	171	0.26	0	34	34	0.05	25
	**10**	658	101	74	175	0.27	6	64	70	0.11	16
	**11**	654.5	73	96	169	0.26	2	84	86	0.13	24
	**12**	666.75	48	41	89	0.13	1	33	34	0.05	24
	**13**	652.75	41	26	67	0.10	1	24	25	0.04	18
	**14**	561.75	70	34	104	0.19	0	32	32	0.06	20
	**15**	160.3	133	24	157	0.98	1	21	22	0.14	14
	**16**	656.25	325	102	427	0.65	5	80	85	0.13	21

Western part of the Gulf of Riga	**17**	654.5	100	439	539	0.82	5	384	389	0.59	34
	**18**	35	92	63	155	4.43	0	57	57	1.63	13
	**19**	651	143	16	159	0.24	0	11	11	0.02	14
	**20**	644	597	46	643	1.00	5	23	28	0.04	0
	**21**	659.75	61	20	81	0.12	0	16	16	0.02	23
	**22**	654.5	650	26	676	1.03	5	14	19	0.03	0
	**23**	663.25	70	19	89	0.13	2	13	15	0.02	14
	**24**	656.25	131	3	134	0.20	1	2	3	0.00	27
	**25**	647.5	482	142	624	0.96	7	98	105	0.16	0
	**26**	656.25	630	281	911	1.39	8	147	155	0.24	9
Southern part of the Gulf of Riga	**28**	570.5	272	147	419	0.73	14	95	109	0.19	35
	**29**	1575	823	2596	3419	2.17	7	1315	1322	0.84	128
	**30**	665	54	55	109	0.16	0	0	0	0.00	6
	**32**	659.75	302	107	409	0.62	4	52	56	0.08	38
	**33**	609	259	134	393	0.65	5	63	68	0.11	29
	**34**	577.5	212	25	237	0.41	1	17	18	0.03	7

Eastern part of the Gulf of Riga	**35**	638.75	141	380	521	0.82	2	337	339	0.53	30
	**36**	649.25	172	48	220	0.34	2	40	42	0.06	5
	**37**	677.25	140	27	167	0.25	0	16	16	0.02	24
	**38**	483	577	172	749	1.55	1	132	133	0.28	33
	**39**	644	263	44	307	0.48	3	41	44	0.07	4
	**40**	721	76	16	92	0.13	2	8	10	0.01	9

Central part of the Gulf of Riga	**41**	656.25	157	67	224	0.34	7	52	59	0.09	25
	**42**	953.75	236	284	520	0.55	11	228	239	0.25	26
	**43**	595	209	32	241	0.41	4	16	20	0.03	15
	**44**	640.5	187	94	281	0.44	12	74	86	0.13	32
	**45**	656.25	260	53	313	0.48	3	28	31	0.05	14
	**46**	651	60	15	75	0.12	6	10	16	0.02	1

The characteristics (shape, size, color) of visually identified microplastic particles verified by chemical composition analysis are expressed as the total number of items found in the specific sample and provided below in the Table 3, Table 4, Table 5. In the cases of classification by size and color, fiber and non-fiber shaped particles are presented separately.

**Table 3:** Classification by shape of the microplastic particles found in the samples.

Sub-region	Station	Fragment	Film	Granule	Bead	Fiber	Foam
Eastern Gotland Basin coastal area	**1**	23	0	0	0	4	0
	**2**	358	32	0	13	4	0
	**3**	45	0	0	3	7	0
	**4**	221	11	0	13	1	1
	**5**	104	2	0	7	3	0
	**6**	11	1	0	2	4	1
	**7**	283	9	0	4	1	7
	**8**	5	0	0	1	0	0

Eastern Gotland Basin offshore	**9**	32	0	0	1	0	1
	**10**	63	0	0	1	6	0
	**11**	78	2	0	3	2	1
	**12**	31	2	0	0	1	0
	**13**	19	1	0	3	1	1
	**14**	31	0	0	1	0	0
	**15**	20	1	0	0	1	0
	**16**	71	8	0	0	5	1

Western part of the Gulf of Riga	**17**	365	9	0	8	5	2
	**18**	53	1	0	3	0	0
	**19**	11	0	0	0	0	0
	**20**	20	3	0	0	5	0
	**21**	15	0	0	1	0	0
	**22**	10	2	2	0	5	0
	**23**	11	0	0	0	2	2
	**24**	2	0	0	0	1	0
	**25**	22	75	0	0	7	1
	**26**	39	106	0	0	8	2

Southern part of the Gulf of Riga	**28**	69	16	2	6	14	2
	**29**	1231	38	0	46	7	0
	**30**	0	0	0	0	0	0
	**32**	50	2	0	0	4	0
	**33**	52	10	0	1	5	0
	**34**	15	0	0	2	1	0

Eastern part of the Gulf of Riga	**35**	315	17	0	3	2	2
	**36**	39	0	0	1	2	0
	**37**	15	1	0	0	0	0
	**38**	126	2	0	3	1	1
	**39**	39	1	0	0	3	1
	**40**	8	0	0	0	2	0

Central part of the Gulf of Riga	**41**	51	0	0	1	7	0
	**42**	216	8	0	3	11	1
	**43**	13	1	0	2	4	0
	**44**	72	1	0	1	12	0
	**45**	23	3	1	1	3	0
	**46**	8	2	0	0	6	0

**Table 4:** Classification by size of the microplastic particles (non-fiber and fiber) found in the samples.

		Fiber					Non-fiber				
Sub-region	Station	≤1mm	1-5mm	5-10mm	10-20mm	>20mm	≤1mm	1-5mm	5-10mm	10-20mm	>20mm
Eastern Gotland Basin coastal area	**1**	2.0	1.0	0.0	1.0	0.0	18	5	0	0	0
	**2**	0.0	3.0	0.0	0.0	1.0	278	113	8	1	3
	**3**	1.0	2.0	1.0	2.0	1.0	35	13	0	0	0
	**4**	0.0	1.0	0.0	0.0	0.0	179	60	6	0	1
	**5**	0.0	1.0	2.0	0.0	0.0	79	32	2	0	0
	**6**	2.0	2.0	0.0	0.0	0.0	9	4	1	0	1
	**7**	0.0	1.0	0.0	0.0	0.0	198	98	7	0	0
	**8**	0.0	0.0	0.0	0.0	0.0	3	3	0	0	0

Eastern Gotland Basin offshore	**9**	0.0	0.0	0.0	0.0	0.0	21	12	1	0	0
	**10**	1.0	2.0	1.0	1.0	1.0	47	17	0	0	0
	**11**	0.0	2.0	0.0	0.0	0.0	57	24	2	1	0
	**12**	0.0	1.0	0.0	0.0	0.0	15	15	3	0	0
	**13**	0.0	1.0	0.0	0.0	0.0	19	5	0	0	0
	**14**	0.0	0.0	0.0	0.0	0.0	27	4	1	0	0
	**15**	0.0	0.0	0.0	0.0	1.0	15	5	1	0	0
	**16**	1.0	4.0	0.0	0.0	0.0	47	30	3	0	0

Western part of the Gulf of Riga	**17**	0.0	3.0	0.0	2.0	0.0	282	98	3	1	0
	**18**	0.0	0.0	0.0	0.0	0.0	42	12	1	0	2
	**19**	0.0	0.0	0.0	0.0	0.0	6	5	0	0	0
	**20**	1.0	4.0	0.0	0.0	0.0	17	6	0	0	0
	**21**	0.0	0.0	0.0	0.0	0.0	14	2	0	0	0
	**22**	0.0	2.0	2.0	1.0	0.0	9	4	1	0	0
	**23**	0.0	0.0	1.0	1.0	0.0	11	2	0	0	0
	**24**	0.0	0.0	0.0	1.0	0.0	1	1	0	0	0
	**25**	1.0	5.0	1.0	0.0	0.0	92	6	0	0	0
	**26**	0.0	7.0	1.0	0.0	0.0	133	13	1	0	0
Southern part of the Gulf of Riga	**28**	1.0	7.0	3.0	3.0	0.0	59	32	4	0	0
	**29**	1.0	5.0	1.0	0.0	0.0	1000	305	5	4	1
	**30**	0.0	0.0	0.0	0.0	0.0	0	0	0	0	0
	**32**	0.0	3.0	1.0	0.0	0.0	22	25	3	2	0
	**33**	0.0	2.0	1.0	1.0	1.0	45	15	2	0	1
	**34**	0.0	1.0	0.0	0.0	0.0	17	0	0	0	0

Eastern part of the Gulf of Riga	**35**	0.0	1.0	1.0	0.0	0.0	254	76	5	1	1
	**36**	0.0	2.0	0.0	0.0	0.0	28	10	0	2	0
	**37**	0.0	0.0	0.0	0.0	0.0	12	3	0	1	0
	**38**	1.0	0.0	0.0	0.0	0.0	100	28	3	1	0
	**39**	0.0	3.0	0.0	0.0	0.0	25	13	3	0	0
	**40**	0.0	1.0	1.0	0.0	0.0	7	0	1	0	0

Central part of the Gulf of Riga	**41**	1.0	1.0	4.0	1.0	0.0	42	10	0	0	0
	**42**	0.0	4.0	2.0	4.0	1.0	147	76	4	1	0
	**43**	0.0	2.0	1.0	1.0	0.0	10	6	0	0	0
	**44**	0.0	10.0	1.0	0.0	1.0	48	25	1	0	0
	**45**	1.0	1.0	0.0	1.0	0.0	18	8	1	1	0
	**46**	0.0	3.0	2.0	0.0	1.0	6	3	1	0	0

**Table 5:** Classification by color of the microplastic particles (non-fiber and fiber) found in the samples.

		Fiber													Non-fiber												
Sub-region	Station	Black	White	Blue	Red	Pink	Purple	Green	Grey	Orange	Yellow	Brown	Transparent	Multicolor	Black	White	Blue	Red	Pink	Purple	Green	Grey	Orange	Yellow	Brown	Transparent	Multicolor
Eastern Gotland Basin coastal area	**1**	0	1	0	0	0	0	3	0	0	0	0	0	0	3	15	1	0	0	0	1	1	0	0	0	0	2
	**2**	0	1	2	1	0	0	0	0	0	0	0	0	0	42	252	18	0	1	3	15	6	1	15	3	30	17
	**3**	1	0	2	0	2	0	2	0	0	0	0	0	0	2	20	9	0	0	0	5	1	0	2	1	3	5
	**4**	1	0	0	0	0	0	0	0	0	0	0	0	0	31	151	15	0	1	0	10	2	1	4	1	12	18
	**5**	1	0	0	0	0	0	2	0	0	0	0	0	0	11	65	5	0	1	0	4	3	4	13	0	1	6
	**6**	1	0	1	0	0	0	1	0	0	0	0	0	1	5	4	5	0	0	0	0	0	0	1	0	0	0
	**7**	0	1	0	0	0	0	0	0	0	0	0	0	0	23	232	11	2	3	4	5	4	1	7	1	3	7
	**8**	0	0	0	0	0	0	0	0	0	0	0	0	0	0	4	0	0	0	0	0	1	0	0	0	1	0

Eastern Gotland Basin offshore	**9**	0	0	0	0	0	0	0	0	0	0	0	0	0	2	22	3	0	0	0	3	0	0	1	0	1	2
	**10**	1	0	0	0	1	0	3	1	0	0	0	0	0	7	44	4	0	0	0	2	0	1	2	0	0	4
	**11**	0	0	0	0	0	0	1	1	0	0	0	0	0	5	55	5	0	0	0	4	3	1	2	0	3	6
	**12**	0	0	1	0	0	0	0	0	0	0	0	0	0	6	22	1	1	0	0	2	1	0	0	0	0	0
	**13**	0	0	0	0	0	0	0	0	0	1	0	0	0	0	11	5	1	1	0	0	1	0	0	0	1	4
	**14**	0	0	0	0	0	0	0	0	0	0	0	0	0	1	12	7	0	1	0	6	2	1	0	1	0	1
	**15**	0	0	0	0	0	0	0	0	0	0	1	0	0	1	14	0	0	2	0	2	0	0	1	0	0	1
	**16**	1	2	0	0	0	0	2	0	0	0	0	0	0	14	43	4	0	0	1	6	5	1	1	0	3	2

Western part of the Gulf of Riga	**17**	2	0	1	0	0	0	1	0	1	0	0	0	0	30	289	11	1	3	0	7	1	1	8	2	18	13
	**18**	0	0	0	0	0	0	0	0	0	0	0	0	0	9	37	4	0	1	0	1	1	0	0	1	1	2
	**19**	0	0	0	0	0	0	0	0	0	0	0	0	0	1	6	1	0	1	0	2	0	0	0	0	0	0
	**20**	1	0	3	1	0	0	0	0	0	0	0	0	0	1	6	5	0	1	0	2	4	0	1	0	1	2
	**21**	0	0	0	0	0	0	0	0	0	0	0	0	0	1	11	1	0	1	0	1	0	0	1	0	0	0
	**22**	1	0	1	1	0	0	1	1	0	0	0	0	0	1	5	1	0	0	0	4	0	0	0	0	3	0
	**23**	2	0	0	0	0	0	0	0	0	0	0	0	0	1	9	1	0	0	0	0	2	0	0	0	0	0
	**24**	0	0	0	0	0	0	0	0	1	0	0	0	0	0	2	0	0	0	0	0	0	0	0	0	0	0
	**25**	3	2	2	0	0	0	0	0	0	0	0	0	0	0	85	0	0	3	0	0	2	0	1	0	7	0
	**26**	5	0	1	0	1	0	0	1	0	0	0	0	0	1	135	3	0	2	0	3	1	0	0	0	1	1
Southern part of the Gulf of Riga	**28**	2	0	5	0	2	0	2	0	0	0	0	3	0	8	43	3	1	6	0	5	3	0	1	0	18	7
	**29**	0	1	2	1	0	0	0	0	0	2	1	0	0	93	963	46	5	19	2	36	39	11	47	13	9	32
	**30**	0	0	0	0	0	0	0	0	0	0	0	0	0	0	0	0	0	0	0	0	0	0	0	0	0	0
	**32**	1	0	1	0	2	0	0	0	0	0	0	0	0	8	6	5	1	4	0	7	9	0	0	1	4	7
	**33**	1	1	1	0	0	0	0	0	0	0	0	1	1	4	37	2	0	2	0	5	1	0	3	0	5	4
	**34**	0	0	0	0	1	0	0	0	0	0	0	0	0	2	9	0	0	0	0	0	1	1	0	0	0	4

Eastern part of the Gulf of Riga	**35**	1	0	1	0	0	0	0	0	0	0	0	0	0	21	271	7	0	7	1	7	7	0	2	0	12	2
	**36**	1	0	1	0	0	0	0	0	0	0	0	0	0	6	27	3	0	1	0	0	1	0	0	0	2	0
	**37**	0	0	0	0	0	0	0	0	0	0	0	0	0	1	14	0	0	0	0	0	1	0	0	0	0	0
	**38**	1	0	0	0	0	0	0	0	0	0	0	0	0	5	101	8	0	1	0	1	3	0	9	0	1	3
	**39**	0	1	0	0	0	0	2	0	0	0	0	0	0	5	27	1	0	0	0	3	1	0	1	0	1	2
	**40**	0	0	2	0	0	0	0	0	0	0	0	0	0	1	7	0	0	0	0	0	0	0	0	0	0	0

Central part of the Gulf of Riga	**41**	2	1	2	0	0	0	1	0	0	1	0	0	0	5	39	3	0	1	0	0	1	0	0	1	0	2
	**42**	2	0	3	0	1	0	1	0	2	0	0	2	0	13	144	12	5	2	0	6	7	2	2	1	10	24
	**43**	0	0	2	0	0	0	1	0	1	0	0	0	0	0	10	1	0	0	0	1	1	0	0	0	0	3
	**44**	2	1	2	0	0	0	5	1	0	1	0	0	0	8	58	1	0	0	0	3	1	0	1	0	2	0
	**45**	2	0	0	0	0	0	0	0	0	1	0	0	0	3	14	4	0	1	0	0	3	0	0	0	3	0
	**46**	1	2	0	0	0	0	0	1	1	0	1	0	0	1	6	1	0	0	0	1	0	0	0	0	1	0

The Table 6 presents information on microplastic particle polymer composition as the total number of items found in the samples. Polymers of fiber and non-fiber shaped particles are given separately.

**Table 6:** Classification by polymer composition of the microplastic particles (non-fiber and fiber) found in the samples.

		Fiber									Non-fiber								
Sub-region	Station	PE	PP	PES	PS	Nylon	Other	Not identified	Organic and salts	Lost	PE	PP	PES	PS	Nylon	Other	Not identified	Organic and salts	Lost
Eastern Gotland Basin coastal area	**1**	3	1	0	0	0	0	0	0	0	21	2	0	0	0	0	0	1	0
	**2**	3	1	0	0	0	0	0	0	0	358	42	0	2	0	1	6	14	1
	**3**	3	4	0	0	0	0	0	0	0	38	9	0	0	0	1	2	3	0
	**4**	0	1	0	0	0	0	1	0	0	218	26	0	2	0	0	6	15	0
	**5**	2	1	0	0	0	0	1	0	0	104	8	0	1	0	0	7	0	0
	**6**	2	0	0	0	1	1	0	0	0	10	2	0	0	0	3	1	2	1
	**7**	0	1	0	0	0	0	0	1	0	261	36	0	6	0	0	11	7	0
	**8**	0	0	0	0	0	0	0	0	0	6	0	0	0	0	0	0	0	0

Eastern Gotland Basin offshore	**9**	0	0	0	0	0	0	0	0	0	25	6	0	3	0	0	3	2	0
	**10**	3	2	0	0	0	1	0	0	0	60	4	0	0	0	0	2	0	0
	**11**	0	1	0	0	1	0	0	0	0	70	12	0	1	0	1	3	0	1
	**12**	0	0	0	0	0	1	0	0	0	30	3	0	0	0	0	0	4	0
	**13**	0	1	0	0	0	0	0	0	0	20	3	0	1	0	0	1	0	1
	**14**	1	0	0	0	0	0	0	0	0	26	6	0	0	0	0	0	0	0
	**15**	0	4	0	0	0	1	0	0	0	18	3	0	0	0	0	0	0	0
	**16**	0	5	0	0	0	0	0	0	0	68	9	1	1	0	1	8	7	0

Western part of the Gulf of Riga	**17**	0	0	0	0	0	0	0	0	0	353	30	0	0	0	1	12	1	3
	**18**	0	0	0	0	0	0	0	0	0	46	10	0	1	0	0	1	4	1
	**19**	0	0	0	0	0	0	0	0	0	6	4	0	0	1	0	2	0	0
	**20**	0	3	2	0	0	0	0	0	0	12	6	2	1	0	2	6	7	0
	**21**	0	0	0	0	0	0	0	0	0	12	4	0	0	0	0	2	1	0
	**22**	1	3	1	0	0	0	1	2	0	13	1	0	0	0	0	4	8	0
	**23**	0	2	0	0	0	0	0	0	0	11	0	0	2	0	0	3	2	0
	**24**	0	0	0	1	0	0	0	0	0	0	2	0	0	0	0	1	0	0
	**25**	1	0	1	1	3	1	7	3	3	90	2	0	4	1	1	11	17	2
	**26**	2	1	0	0	0	5	1	1	0	102	6	1	3	0	35	19	36	1
Southern part of the Gulf of Riga	**28**	1	11	0	0	0	2	0	0	0	73	17	1	1	0	3	9	8	0
	**29**	0	7	0	0	0	0	1	1	0	1126	175	0	4	0	10	49	44	8
	**30**	0	0	0	0	0	0	0	0	0	0	0	0	0	0	0	0	0	0
	**32**	0	4	0	0	0	0	0	0	0	26	21	0	3	0	2	18	3	6
	**33**	0	2	2	0	1	0	0	0	0	52	9	0	0	1	1	4	2	2
	**34**	1	0	0	0	0	0	0	0	0	14	2	0	1	0	0	0	1	0

Eastern part of the Gulf of Riga	**35**	0	2	0	0	0	0	0	0	0	297	37	0	3	0	0	10	10	0
	**36**	2	0	0	0	0	0	0	0	0	35	4	0	0	0	1	0	1	0
	**37**	0	0	0	0	0	0	0	0	0	15	1	0	0	0	0	0	2	0
	**38**	1	0	0	0	0	0	0	0	0	116	15	0	1	0	0	15	3	0
	**39**	3	0	0	0	0	0	0	0	0	36	4	0	1	0	0	1	1	0
	**40**	0	2	0	0	0	0	0	0	0	7	1	0	0	0	0	3	0	0

Central part of the Gulf of Riga	**41**	2	3	0	0	1	1	0	3	0	42	9	0	0	0	1	6	3	0
	**42**	1	10	0	0	0	0	0	0	2	191	29	0	1	0	7	7	3	8
	**43**	0	3	0	0	0	1	0	0	0	14	2	0	0	0	0	5	0	1
	**44**	6	6	0	0	0	0	0	0	0	63	11	0	0	0	0	8	2	0
	**45**	0	3	0	0	0	0	0	0	0	23	4	0	0	0	1	2	3	4
	**46**	1	3	0	0	0	2	0	0	0	7	3	0	0	0	0	1	1	0

The negative controls in three-replicate proved background contamination by fibers during samples treatment (blank samples) to be 7, 12 and 23 fibers per blank sample. The positive controls of particles in the three-replicate spike-and-recovery test showed recovery rates of 90%, 95% and 95%.

## 3 Experimental Design, Material and Methods

### 3.1 Sampling

Altogether 44 micro debris samples (28 in Gulf of Riga and the 16 Eastern Gotland Basin) were collected. Sampling occurred over the time period from May to September 2018 during 7 cruises to cover coastal and open waters of the study area representatively. The samples were taken from the State Environmental Service (SES) fishing control and marine monitoring vessel “MARE” and Tallinn University of Technology research vessel “SALME”.

Sampling was performed by deploying Manta net (HydroBios, mesh size 300 µm, frame opening area 0.28 m^2^, length 3 m) at 7 m distance from the side of the vessel and trawled for 1 hour at the speed of approximately 2 knots, following the recommendations of Gago et al., 2018 [2], Hydro-bios Apparatebau GmbH, 2017 [3] and Viršek et al. 2016 [5]. The meteorological parameters were recorded immediately before sampling, using devices available on the sampling vessel – anemometer and wind vane for detection of wind speed and direction, thermometer for determining air temperature, and online weather data portal “Windy” [4] to find out current wave height. The start coordinates and time was recorded at the moment when the net was placed in the water, and end coordinates and time – when the net was raised from the water. Trawling duration and distance were altered (reduced) in cases when surface water algal blooms caused clogging of the net. The volume of water filtered through the net was calculated by multiplying transect distance (determined by coordinates) with the average submerged opening area of the trawl (0.175 m^2^). The average sample volume was 639.1 ±197.4 m^3^ (min 35 m^3^ at station No. 18; max 1575 m^3^ at station No. 29).

After sampling, the net was rinsed from the outside with sea water to concentrate the sample at the cod end of the net and exclude the possibility of contamination. Then, the cod end was removed, and its content was rinsed into previously decontaminated stainless-steel sieve (mesh size 200 µm, ⌀ 100 mm, RETSCH production) using filtered distilled water. To facilitate sample treatment process better, largest non-synthetic origin particles that were easily visually recognizable, such as leaves, branches, feathers, marine organisms, insects etc. were discarded by first picking them up with tweezers and thereafter rinsing them over the steel sieve with filtered distilled water in order to retain any micro debris particles that could have been attached to them. Finally, the sample from the sieve was transferred to previously decontaminated glass jar by rinsing with filtered distilled water. The jar opening was then covered with aluminum foil and a metal lid, and frozen at the temperature of -4 ±2°C until sample treatment.

### 3.2 Quality assurance

In order to minimize external sample contamination, several precautionary measures were taken while working with the samples. During sampling, the Manta net was towed from the side of the vessel to avoid airborne or waterborne vessel-origin contamination. Only glass or metal equipment was used for samples handling, storage and treatment process. All equipment that came in contact with samples was thoroughly rinsed before use with filtered distilled water. All reagents used for samples treatment were filtered through glass fiber (GF) filters (equivalent to pore size 1.2 µm, ⌀ 47 mm, Whatman®) before use. Cotton or linen laboratory coats with specific color (green or purple) were used to maximize capacity to determine airborne contamination from clothing during samples handling and treatment. Nitrile gloves were worn throughout the treatment process. The polymer spectrum of nitrile gloves and Manta net was taken, and corresponding polymer types were excluded from data set after samples polymer analysis. Sample treatment was done in fume hood and samples were covered with aluminum foil at all times when not worked with or when placed in the shaking water bath. To minimize possible particle loss, the same thoroughly rinsed beaker was used for each treatment step of the respective sample.

Three procedural blank samples were run to assess the level of background contamination degree in the laboratory by applying the same processing steps as for the samples. For estimation of airborne contamination during visual analysis, for each sample separate Whatman glass fiber filter in Petri dish was exposed next to the microscope in the proximity of sample.

Spike-and-recovery approach in triplicate was used to assess the potential non-fiber particle loss during sample treatment with standardized polystyrene beads (⌀ 100 µm density 1.05 g/cm^−3^ 100 µm size) of red color (Sigma-Aldrich product no. 56969-10ML-F). Each of the positive control samples contained 100 beads in distilled water and was treated in the same manner as field samples.

### 3.3 Sample treatment

**Fig. 2:**
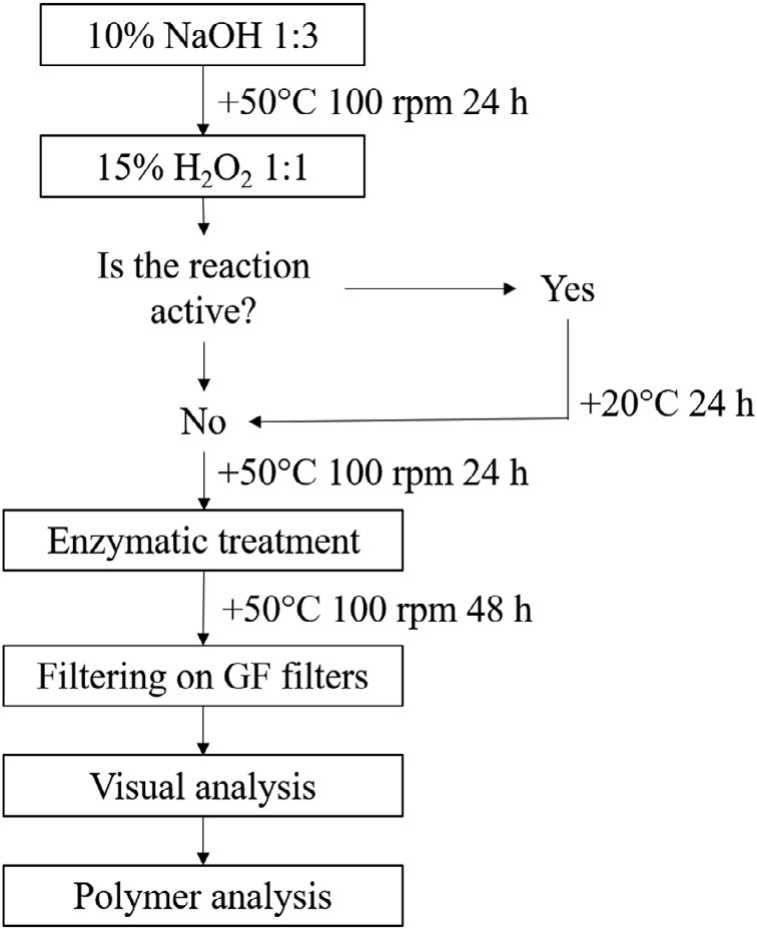
Sample treatment and analysis protocol.

To remove biological material from the sample, several step procedure was conducted (Fig. 2) based on the protocol described by Gago et al. 2018 [2]. Before the treatment, samples were thawed and transferred to a glass beaker using filtered distilled water. For the first treatment step alkaline digestion method was used, adding 10% sodium hydroxide (NaOH, Firma Chempu) to the sample in proportion 1:3 (1/4 of final volume sample and 3/4 NaOH). Then the samples were covered with foil and incubated in a shaking water bath for 24h at the temperature of +50°C and shaking frequency 100 rpm. After that, the NaOH solution was removed by filtering the samples through stainless steel sieve (mesh size 200 µm) and thoroughly rinsing the beaker with filtered distilled water. Then samples from the sieve were transferred to the previously used beaker by rinsing with filtered distilled water. For the second treatment step oxidative digestion method was used by adding 30% hydrogen peroxide (H_2_O_2_, Carl Roth) to the sample in proportion 1:2 (1/3 of the final volume sample, 2/3 H_2_O_2_), covering the sample with foil. If the oxidation reaction was active, the sample was kept in fume hood for 24h at room temperature. If the oxidation reaction was not active or if 24h at room temperature had passed, the samples were incubated in a shaking water bath for 24h at the temperature of +50°C and shaking frequency 100 rpm. After that, the H_2_O_2_ solution was removed by filtering the samples through stainless steel sieve (mesh size 200 µm) and thoroughly rinsing the beaker with filtered distilled water. Then samples from the sieve were transferred to the previously used beaker by rinsing with 100 mL of acetate buffer (pH 4.8). For the last treatment step, enzymatic digestion method was used by adding acetate buffer until 300 mL mark, and 0.5 mL cellulase (Cellulase enzyme blend, activity >1000 U/mL, Sigma Aldrich) and 0.5 mL viscozyme (Viscozyme L. cellulolytic enzyme mixture, activity >100 FBGU/g, Sigma-Aldrich) to the sample. Then the samples were covered with foil and incubated in a shaking water bath for 48h at the temperature of +50°C and shaking frequency 100 rpm. After that, the acetate buffer solution was removed by filtering the samples through stainless steel sieve (mesh size 200 µm) and thoroughly rinsing the beaker with filtered distilled water. If after this step a considerable amount of microcrustaceans was present in the samples, the samples were transferred from the sieve to the previously used beaker by rinsing with 100 mL of acetate buffer (pH 4.8), later fixing the buffer volume until 300 mL mark and adding 0.1-0.7 mL chitinase (activity >100 U/mL, ASA Spezialenzyme GmbH). Then the samples were covered with foil and incubated in a shaking water bath for 48h at the temperature of +37°C and shaking frequency 100 rpm. After enzymatic treatment, acetate buffer solution was removed by filtering the samples through stainless steel sieve (mesh size 200 µm) for the last time, thoroughly rinsing the beaker with filtered distilled water. Then samples were filtered on GF filters. In cases when the described sample treatment procedure was not sufficient to remove all organic material from the samples, a series of several GF filters were used.

### 3.4 Sample visual analysis

**Fig. 3:**
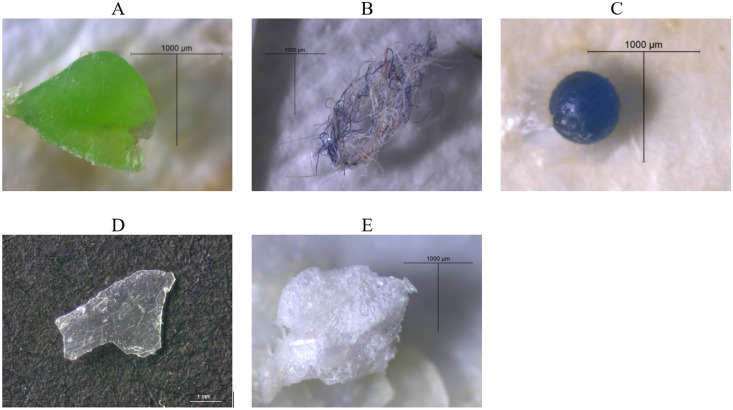
Example of microplastic particles shape. A – fragment; B – fiber; - C – bead; D – film; E – foam.

Particle visual identification was performed by using the automated upright microscope system Leica DM400 B LED equipped with digital microscope color camera DFC 295 and microscope software platform Leica Application Suite V4.1. At the same time particle morphology was registered – all potential microplastic particles on the GF filters were counted and characterized by their visual features such as shape, size and color, and photographed. Particles were categorized into five types according to their shape (Fig. 3) similarly as done by Gago et al. 2018 [2] and Viršek et al. 2016 [5]. Category A is fragments that have irregular form, thickness, sharp or crooked edges as well as variety of different colors. Category B is fibers that are supple, can have different colors and lengths but width in relation to length is considerably smaller. Category C is beads that have regular round shape, diverse colors and are usually smaller than 1 mm in diameter. Category D is films that also have irregular form and different colors, but in comparison to fragments, they are thin, flexible and usually transparent. Category E is foams that are soft, with irregular form and usually white or yellow color.

The size of particles was determined by measuring the length (longest dimension) and width (longest perpendicular dimension of length) of each particle. Particles classification by size was done by affiliating them to one of five defined size classes – small microplastics (≥0.3 to ≤1 mm), large microplastics (>1 to ≤5 mm), small mesoplastics (>5 to ≤10 mm), large mesoplastics (>10 to ≤20 mm) and macroplastics (>20 mm). The size classes were adapted from those used by Cole et al. 2011 [6], HELCOM 2015 [7], Isobe et al. 2014 [8] and Iwasaki et al. 2017 [9].

In regard to the color of particles, they were classified as transparent/translucent, black, white, blue, red, pink, purple, green, grey, orange, yellow, brown or multi-colored.

### 3.5 Polymer composition analysis

During visual analysis particles in sizes suitable for manual handling were individually transferred with tweezers from GF filters into vial plates for further polymer type identification. Analysis was done applying Attenuated Total Reflection Fourier-Transform Infrared (ATR-FTIR) spectroscopy method using Thermo Fisher Scientific Nicolet iS20 FTIR spectrometer combined with Thermo Fisher Scientific OMNIC computer software. The particles were manually transferred from vial plates to ATR-FTIR where they were one by one fixed to a cleaned diamond crystal and, for the acquisition of polymer spectra, scanned with a resolution of 4 cm^−1^ and an IR range of 4000-400 cm^−1^. The acquired spectra were automatically compared to reference spectra in 30 spectral libraries that contained more than 15 000 spectra of both synthetic and natural materials and compounds. If the compatibility was higher than 80%, the match was considered to be credible, but if it was lower than 80% and higher than 60%, the compatibility was critically evaluated by manually comparing the distinctive spectral peaks of particles spectra to the best matches offered from spectral libraries. Compatibility below 60% was not considered and was recognized as invalid.

## Ethics Statements

This study did not involve human or animal subjects, and no data from social media platforms were used.

## CRediT authorship contribution statement

**Marta Barone:** Methodology, Investigation, Data curation, Writing – original draft. **Natalija Suhareva:** Methodology, Data curation, Visualization, Writing – review & editing. **Juris Aigars:** Funding acquisition, Project administration, Writing – review & editing. **Ieva Putna-Nimane:** Conceptualization, Methodology, Supervision. **Inta Dimante-Deimantovica:** Methodology, Writing – review & editing.

## Declaration of Competing Interest

The authors declare that they have no known competing financial interests or personal relationships that could have appeared to influence the work reported in this paper.

## Data Availability

Dataset on microplastic concentrations, characteristics, and chemical composition in the marine surface waters of Latvia – the Eastern Gotland basin and the Gulf of Riga (Original data) (Mendeley Data).
